# Effect of dietary copper addition on lipid metabolism in rabbits

**DOI:** 10.1080/16546628.2017.1348866

**Published:** 2017-07-06

**Authors:** Liu Lei, Sui Xiaoyi, Li Fuchang

**Affiliations:** ^a^ Shandong Provincial Key Laboratory of Animal Biotechnology and Disease Control and Prevention, Shandong Agricultural University, Taian, China

**Keywords:** Copper, lipid metabolism, rabbits, AMPK, PPAR

## Abstract

The present study was conducted to investigate the effect of copper supplementation on lipid metabolism in rabbits. Our study showed dietary copper addition (5-45 mg/kg) increased body mass gain, but decreased fat and liver weights compared with those in the control group (*P* < 0.05). Copper (45 mg/kg) addition significantly increased the skeletal muscle weight, but inhibited cytoplasmic lipid accumulation in liver, skeletal muscle and adipose tissue (*P* < 0.05). Compared with the control group, dietary copper addition (45 mg/kg) significantly increased plasma triglyceride levels but decreased very low density lipoprotein levels (*P* < 0.05). Copper treatment significantly increased gene expression of carnitine palmitoyltransferase (CPT) 1, CPT2 and peroxisome proliferator-activated receptor (PPAR) a in liver (*P* < 0.05). In skeletal muscle, CPT1, CPT2, fatty acid transport protein, fatty acid-binding protein, and PPARa mRNA as well as phosphorylated AMP-activated protein kinase (AMPK) levels were significantly up-regulated by copper treatment (*P* < 0.05). Rabbits receiving copper supplementation had higher CPT1, CPT2, PPARa and hormone-sensitive lipase mRNA levels in adipose tissue (P < 0.05). In conclusion, copper promoted skeletal muscle growth and reduced fat accretion. PPARa signaling in liver, skeletal muscle and adipose tissues and AMPK signaling in skeletal muscle tissue were involved in the regulation of lipid metabolism by copper.

## Introduction

Fat is the largest energy reserve in mammals. Most tissues are involved in fatty acid metabolism, but three are quantitatively more important than others: adipose tissue, skeletal muscle, and liver tissue. Each of these tissues has a triglyceride store that can be hydrolyzed (mobilized) in a regulated way to release fatty acids. In the case of adipose tissue, these fatty acids may be released into the circulation for delivery to other tissues. In the case of muscle tissue, fatty acids are a substrate for oxidation. In the case of liver tissue, they are a substrate for re-esteriﬁcation within the endoplasmic reticulum to produce triglycerides secreted as very low density lipoprotein (VLDL) [1,2]. The excessive accumulation of triglycerides in the skeletal muscle and liver is sometimes called ectopic fat deposition.

Many regulating factors and pathways are involved in the utilization of fatty acids. Acetyl-CoA carboxylase (ACC), fatty acid synthase (FAS), and carnitine palmitoyltransferase (CPT) are the key regulators of fatty acid synthesis and oxidation [3]. The lipoprotein lipase (LPL) and hormone-sensitive lipase (HSL) enzymes are rate-limiting steps for the turnover of fatty acids in adipose tissue [4]. The fatty acid transport protein (FATP) and the fatty acid-binding protein (FABP) promote the cellular uptake and transport of fatty acids [5]. AMP-activated protein kinase (AMPK), a positive regulator of intracellular fatty acid metabolism, is activated by an increase in the AMP:adenosine triphosphate (ATP) ratio [6]. The activation of AMPK stimulates energy-generating pathways in several tissues while inhibiting energy-consuming pathways. AMPK activation promotes fatty acid oxidation and glucose uptake in skeletal muscle. Conversely, in the liver, AMPK activity inhibits fatty acid and cholesterol synthesis. Lipogenesis is also reduced in adipose tissue by AMPK activation [7]. Peroxisome proliferator-activated receptors (PPARs) belong to a family of nuclear transcription factors that function in a ligand-dependent manner. To date, three different PPAR isoforms – α, β, and γ – and splice variants have been identified that are encoded by separate genes [8]. The tissue-specific expression pattern of these transcription factors is indicative of their function in those tissues [8]. PPAR-α is involved in fatty acid catabolism (β and ω oxidation pathways) and is most abundant in the liver, adipose tissue, and skeletal muscle [9]. PPAR-γ is the most highly restricted in its expression pattern. Its primary sources are adipose, macrophage, and mammary tissue [9,10]. PPAR-γ is required for the differentiation of adipose tissue in vivo and in vitro [11]. Some of the genes regulated by PPAR-γ in adipose tissue include adipocyte fatty acid-binding protein, LPL, acyl-CoA synthetase, stearoyl-CoA synthetase, and phosphoenolpyruvate carboxykinase [12]. In addition, lipid metabolism can be regulated by hormones (e.g. insulin and leptin) [13,14] and nutritional state (carbohydrates and copper) [15,16].

Copper is an essential element for most living organisms and plays an important role in aquatic physiological processes. Dietary copper deficiency is associated with a variety of metabolic changes, including hypercholesterolemia, increased blood pressure, and glucose intolerance, in ducks [17], laying hens [18], and rodents [19,20]. The effect of copper on lipid metabolism has been disputed in previous studies. Copper addition decreased the total cholesterol concentrations in serum [21,22]. Copper supplementation given to Simmental steers had no effect on lipid or cholesterol metabolism [23]. Copper exposure increased lipogenesis and fatty acid uptake [16]. Zhu et al. [24] found that the effect of copper on lipid metabolism in hepatocytes was concentration and time dependent in vitro. In general, studies on the effect of copper on lipid metabolism have focused on plasma and liver tissue, but these results need to be further tested. Furthermore, the mechanism of copper regulation of lipid metabolism as a whole is still unclear.

Obesity and obesity-associated disease (e.g. hepatic steatosis) are becoming global health problems in adults and children [25]. The present study investigated the effect of dietary copper addition on the lipid metabolism and related signaling pathway (AMPK and PPARs) in the liver, skeletal muscle, and adipose tissue of rabbits and determined the possibility of copper-induced attenuation of excess lipid accumulation.

## Materials and methods

### Animals

Rex rabbits (30 days old) were individually housed in self-made plastic cages (60 cm × 40 cm × 40 cm). The temperature and lighting were maintained according to commercial conditions. The diets were formulated following de Blas and Mateos [26], and food was pelleted using pressure. The diameter of the pellets was 4 mm. The ingredients and composition of diet are listed in Supplementary Table S1. All rabbits had free access to food and water during the rearing period. Copper addition was in form of copper sulfate pentahydrate (Zouping Runxin Chemical Co. Ltd., Heze, China). This study was approved by the Shandong Agricultural University and was conducted in accordance with the Guidelines for Experimental Animals of the Ministry of Science and Technology (Beijing, China).

### Experimental protocol and sample collection

At 30 days of age, 144 rabbits of similar body weight (1072 ± 15 g) were divided into four groups (36 replicates per group and one rabbit per replicate). The groups were fed a basal diet (control; measured copper content 8.19 mg/kg) or a basal diet supplemented with 5, 15, or 45 mg/kg copper (measured copper content 13.59, 23.78, and 54.08 mg/kg, respectively). The experiment lasted for 8 weeks, including a 1-week adaptation period and a 7-week experimental period. At the end of the trial, 32 rabbits (8 rabbits per group, half male and half female, with an average body weight equal to that of the entire treatment group) were electrically stunned (70 V, pulsed direct current, 50 Hz for 5 s), and 10 mL of blood was collected immediately from the heart. Plasma was obtained following centrifugation at 400 *g* for 10 min at 4°C and stored at −20°C for subsequent analysis. The rabbits were sacrificed by cervical dislocation, and the liver, skeletal muscle, and adipose tissue were harvested and weighed. According to the effects of copper administration on lipid accumulation, the 45 mg/kg doses of copper (measured copper content 54.08 mg/kg) were chosen for the subsequent experiments. A 1–2 g sample of liver, shoulder fat, and thigh muscle from the control group and 45 mg/kg doses from copper treatment group (total copper content of diet 54.08 mg/kg) was obtained, cooled in liquid nitrogen, and stored at −70°C for gene and protein expression analysis.

### Measurements

Plasma VLDL concentration was determined using the method described by Barter and Lally [27]. Plasma triglyceride concentration was measured spectrophotometrically with commercial diagnostic kits (Hitachi High-Technologies Corp., Jiancheng Bioengineering Institute, Nanjing, PR China). Plasma leptin concentration was determined using a validated sandwich enzyme-linked immunosorbent assay (Uscn Life Science, Inc., Wuhan, China) with a leptin-specific antibody. The intra-assay coefficient of variation was 2.0%. Plasma insulin was measured by radioimmunoassay with a guinea pig anti-porcine insulin serum (3V Bio-engineering group Co., Weifang, PR China). The intra-assay coefficient of variation was 2.04%.

The dietary copper content was determined by atomic absorption spectrophotometry according to the description of Allan [28]

The accumulation of cytoplasmic lipid droplets was visualized by Oil Red O staining according to the protocol of Lillie and Fullmer [29]. Brieﬂy, tissues were immediately frozen in liquid nitrogen and cut using a Leica CM-1850 cryostat microtome (Leica, Wetzlar, Germany). Afterwards, 16-mm-thick sections were ﬁxed in 4% formaldehyde for 10 min and stained with ﬁltered 0.5% Oil Red O (Sigma–Aldrich, St Louis, MO), which was dissolved in isopropyl alcohol, for 15 min at room temperature. Morphometric analysis was performed on 10 randomly chosen ﬁelds containing transverse sections of liver, muscle, and fat from each rabbit. The selected ﬁelds were photographed using an Olympus CX41 phase contrast microscope (Olympus, Tokyo, Japan). The volume density of each Oil Red O-positive liver, muscle, and fat tissue was determined using the point-counting method described by Weibel and Bolender [30].

Total RNA extraction and quantitative reverse transcription polymerase chain reaction (PCR) were performed, as described previously [31]. Primer sequences are shown in Supplementary Table S2. The PCR data were analyzed using the 2^−ΔΔCT^ method [32]. The mRNA levels of target genes were normalized to those of glyceraldehyde 3-phosphate dehydrogenase (GAPDH) and β-actin (ΔCT). Based on the Ct values, GAPDH and β-actin mRNA expression were stable across treatments in this study (*p* > 0.1).

The protein concentration was determined using a BCA assay kit (Beyotime, Jiangsu, China). After boiling at 100ºC, the protein extracts (30 μg) were electrophoresed in 7.5–10% SDS polyacrylamide gels following the procedure described by Laemmli [33]. The separated proteins were then transferred onto nitrocellulose membranes at 100 V at 4ºC. The membranes were stripped to assay phosphorylated proteins and actin separately. The membranes were blocked and immunoblotted with two primary antibodies: phospho-AMPKαTh^r172^ antibody and AMPKα antibody. Protein detection was performed using horseradish peroxidase–labeled goat anti-mouse immunoglobulin G (H + L) secondary antibody by enhanced chemiluminescence with Western blotting detection reagents (Beyotime). Monoclonal mouse anti-tubulin antibody was used as a loading control. Western blots were developed and quantified with BioSpectrum 810 Imaging System using VisionWorksLS 7.1 software (UVP LLC, Upland, CA).

### Statistical analysis

The data were presented as the means ± standard error of the mean (SEM). Data from more than two groups were analyzed by analysis of variance (ANOVA), followed by Tukey’s HSD or Dunnett’s multiple comparisons. Data from two groups were analyzed by Student’s *t*-test. All statistical analyses were performed with the JMP Pro software (SAS Institute, Cary, NC). Before ANOVA or the *t*-test was performed, data were analyzed for homogeneity of variances. For food intake and body mass gain analysis, *n* = 36; for tissue and organ weight analysis, *n* = 8; for cytoplasmic lipid droplets content analysis, *n* = 8; for all mRNA levels analysis, *n* = 8; and for protein expression analysis, *n* = 8. Differences at *p* < 0.05 were considered signiﬁcant.

## Results

### Animal growth, tissue development and lipid droplet deposition

Compared with control rabbits, copper had no signiﬁcant effect on feed intake (*p* > 0.05; Table 1). However, copper addition (15–20 mg/kg) induced an increase in body mass gain (*p* < 0.05). The weight or ratio liver, shoulder fat, and perirenal fat were significantly decreased by copper treatment compared to the control group (Table 1). The dietary addition of copper (45 mg/kg) significantly decreased the ratio and yield of perigastric fat compared to the control group (*p* < 0.05) but significantly increased the skeletal muscles (foreleg muscle and hindleg muscle) ratio and yield (*p* < 0.05).

**Table 1.** Effect of copper treatment on body mass gain (*n* = 36), liver growth, skeletal muscle growth, and lipid accumulation (*n* = 8) in rabbits

	0	5	15	45	*p*
Body mass gain (g/day)	22.90 ± 0.49^b^	24.43 ± 0.44^ab^	25.10 ± 0.30^a^	24.79 ± 0.53^a^	0.0455
Food intake (g/day)	124.10 ± 1.01	124.39 ± 1.30	126.68 ± 0.7	124.25 ± 0.80	0.2802
Liver yield (g)	100.72 ± 2.30^a^	86.46 ± 4.73^bc^	89.98 ± 4.25^b^	72.56 ± 2.33^d^	＜0.0001
Liver yield/BW (%)	3.78 ± 0.10^a^	3.28 ± 0.15^b^	3.42 ± 0.15^b^	2.82 ± 0.09^c^	0.0001
Foreleg yield (g)	144.99 ± 4.64^b^	151.44 ± 6.40^ab^	150.75 ± 3.93^ab^	158.41 ± 3.31^a^	0.0394
Hindleg yield (g)	322.10 ± 10.82^b^	333.35 ± 11.72^ab^	337.10 ± 7.50^ab^	357.10 ± 5.59^a^	0.0419
Foreleg yield/BW (%)	5.43 ± 0.14^b^	5.74 ± 0.11^b^	5.73 ± 0.12^b^	6.16 ± 0.10^a^	0.0072
Hindleg yield/BW (%)	12.08 ± 0.35^b^	12.65 ± 0.18^b^	12.83 ± 0.27^b^	13.89 ± 0.25^a^	0.0024
Shoulder fat yield (g)	7.32 ± 0.50^a^	5.50 ± 0.54^bc^	5.21 ± 0.28^c^	5.63 ± 0.23^bc^	0.0063
Perirenal fat yield (g)	34.58 ± 4.14^a^	23.56 ± 3.10^b^	24.75 ± 1.15^b^	23.40 ± 2.20^b^	0.0367
Perigastric fat yield (g)	9.90 ± 1.18^a^	8.65 ± 1.02^ab^	7.63 ± 0.91^ab^	6.40 ± 0.56^b^	0.0974
Shoulder fat yield/BW (%)	0.27 ± 0.01^a^	0.20 ± 0.02^b^	0.19 ± 0.01^b^	0.21 ± 0.01^b^	0.0024
Perirenal fat yield/BW (%)	1.27 ± 0.14^a^	0.92 ± 0.12^b^	0.92 ± 0.05^b^	0.91 ± 0.08^b^	0.0650
Perigastric fat yield/BW (%)	0.39 ± 0.04^a^	0.30 ± 0.03^ab^	0.28 ± 0.03^b^	0.27 ± 0.02^b^	0.0700

Values are the means ± standard error of the mean (for food intake and body mass gain, *n* = 36; for ratio and yield of tissue or organ, *n* = 8); Means with different superscripts (a, b, c, and d) are significantly different (*p* < 0.05).

**Figure 1.**
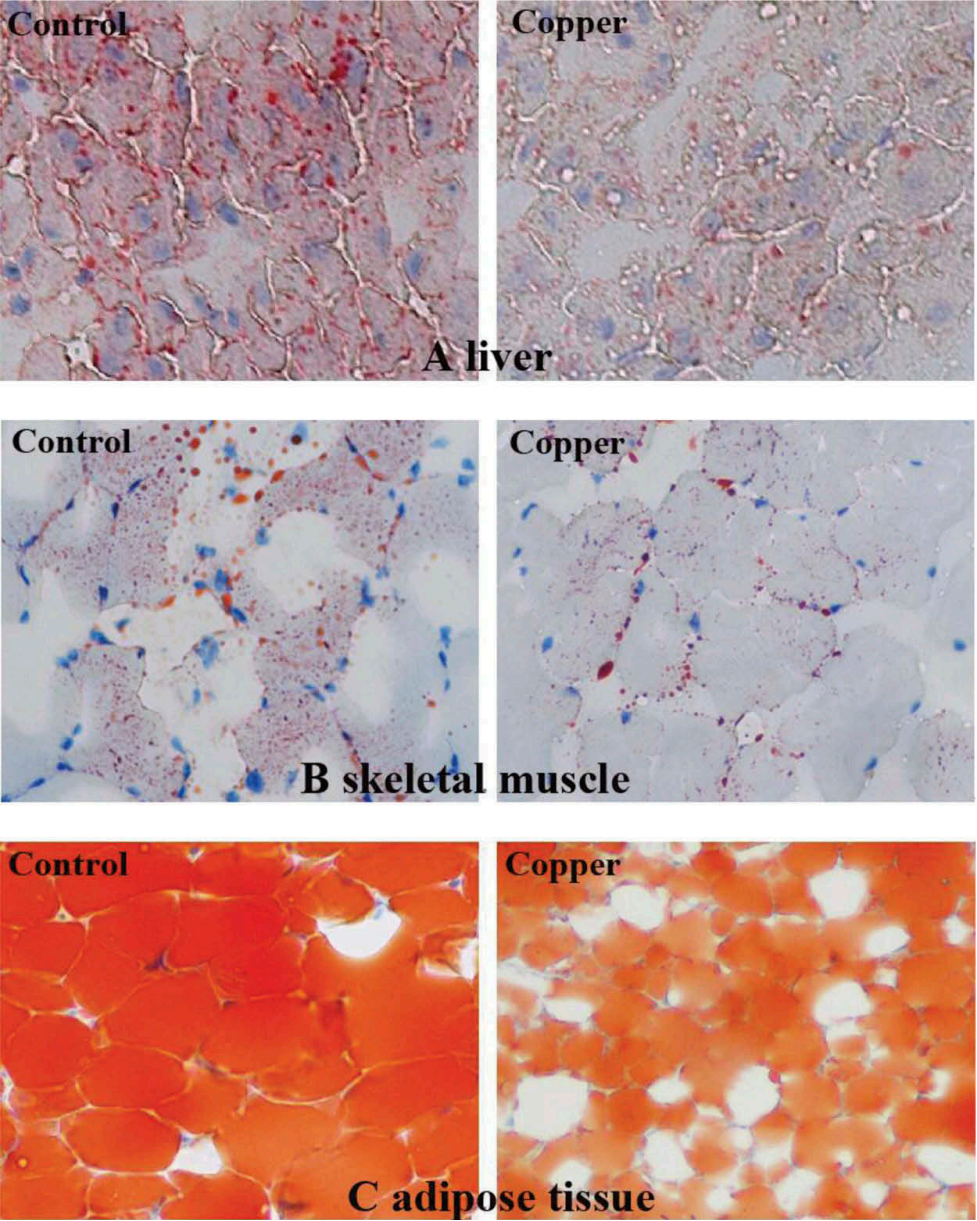
Oil Red O staining of cytoplasmic lipid droplets showing the effect of dietary copper treatment (45 mg/kg) on lipid accumulation in the liver (a), skeletal muscle (b), and adipose tissue (c) from rabbits (*n* = 8; 200×).

The dietary copper treatment significantly inhibited cytoplasmic lipid accumulation in the liver, skeletal muscle, and adipose tissue compared to the control group (*p* < 0.05; Figure 1). Furthermore, copper addition significantly decreased the size of adipocytes compared to the size of those in the control group (*p* < 0.05).

### Plasma parameters

**Figure 2.**
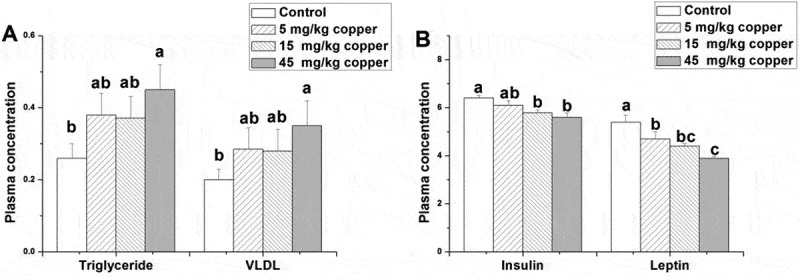
Effects of dietary addition of copper on plasma concentrations of triglycerides (mmol/L), very low density lipoprotein (absorbance), insulin (uIU/mL), and leptin (uIU/mL) in rabbits. Values are the means ± standard error f the mean (SEM; *n* = 8). Means with different superscripts (a–c) are significantly different (*p* < 0.05).

The associated hormone and fat metabolite concentrations in plasma were determined, and the results are shown in Figure 2. Copper treatment (45 mg/kg) significantly increased plasma triglycerides compared to the control group (*p* < 0.05; Figure 2a). In contrast, the dietary addition of copper (45 mg/kg) significantly decreased plasma level of VLDL (*p* < 0.05; Figure 2a). Compared ti the control, the dietary addition of copper (5–45 mg/kg) significantly decreased the plasma leptin level (*p* < 0.05; Figure 2b). Besides, the rabbits in the 15 and 45 mg/kg copper supplied groups had a lower level of plasma insulin than the control did (*p* < 0.05; Figure 2b).

**Figure 3.**
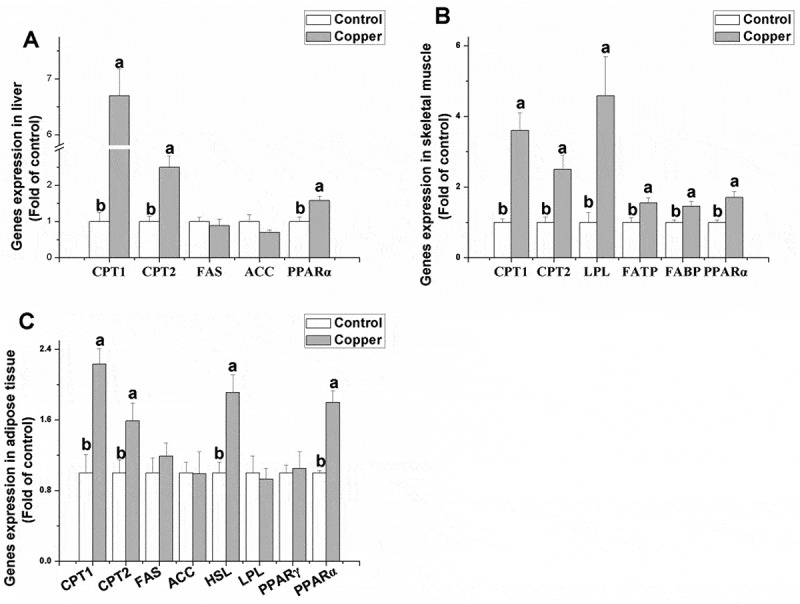
Effects of dietary copper treatment (45 mg/kg) on relative gene expression in lipid metabolism in the liver, skeletal muscle, and adipose tissue. Values are the means ± SEM (*n* = 8). Means with different superscripts (a and b) are significantly different (*p* < 0.05).

**Figure 4.**
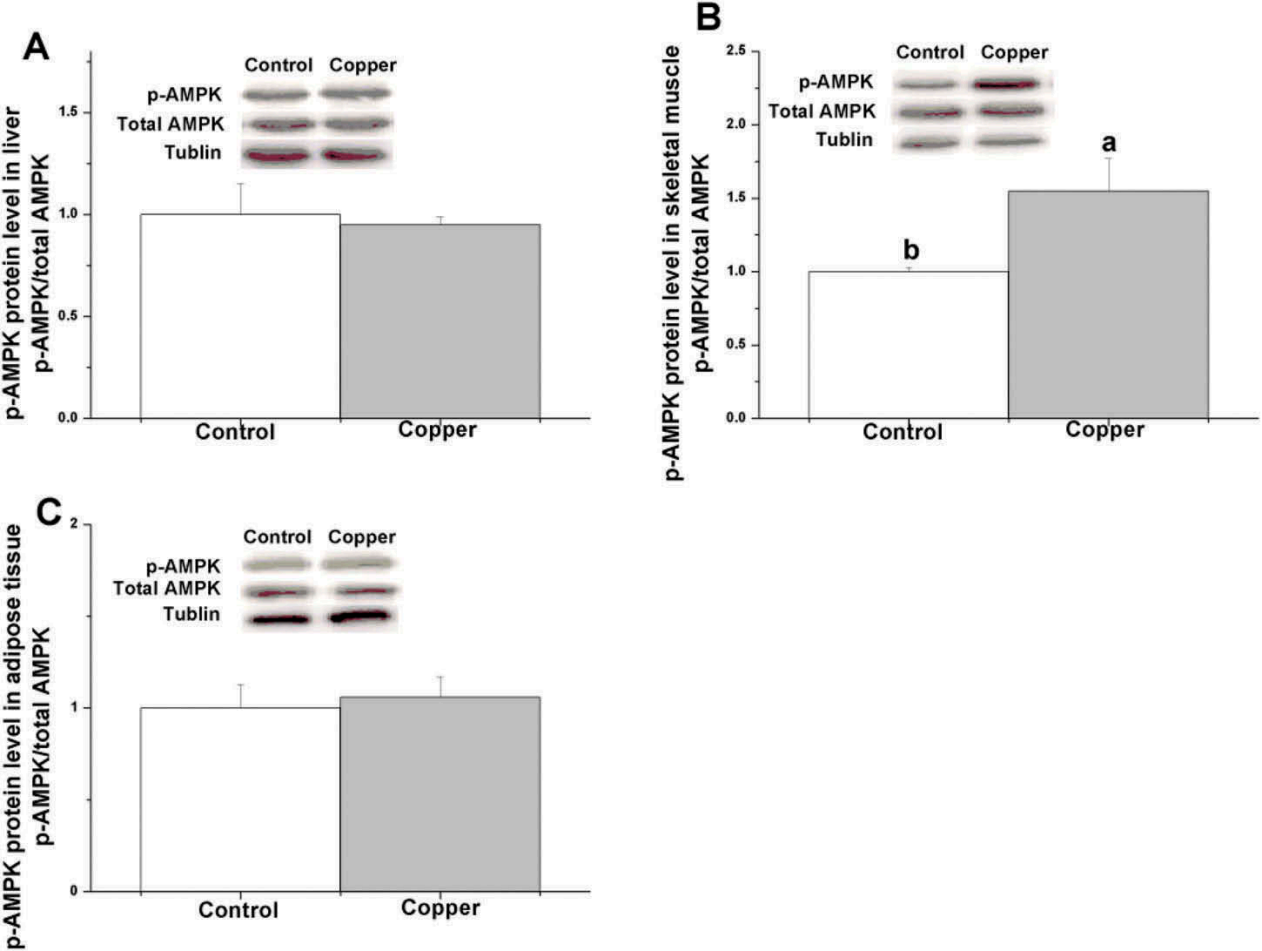
Effects of dietary copper treatment (45 mg/kg) on phosphorylated AMP-activated protein kinase protein level in the liver, skeletal muscle, and adipose tissue. Values are the means ± SEM (*n* = 8). Means with different superscripts (a, b) are significantly different (*p* < 0.05).

### Enzyme mRNA and protein levels

Through determining relative gene expression in lipid metabolism (Figure 3), it was found that the dietary addition of copper significantly increased gene expression of CPT1, CPT2, and PPAR-γ in the liver (*p* < 0.05) but did not affect gene expression of FAS and ACC (*p* > 0.05). In the skeletal muscle, gene expression of CPT1, CPT2, LPL, FATP, FABP, and PPAR-α was significantly upregulated by copper treatment (*p* < 0.05). Compared to the control group, copper treatment had no signiﬁcant effect on the mRNA levels of FAS, ACC, LPL, and PPAR-γ in the adipose tissue (*p* > 0.05). In contrast, rabbits receiving the copper supplement had signiﬁcantly higher mRNA levels of CPT1, CPT2, HSL, and PPAR-α in the adipose tissue compared to the control group (*p* < 0.05).

Figure 4 shows that dietary copper treatment significantly increased the p-AMPK protein level in skeletal muscle tissue (*p* < 0.05) but not in the liver or adipose tissue (*p* > 0.05).

## Discussion

This study demonstrated for the first time the effect of copper addition on lipid metabolism as a whole in rabbits. It showed that (1) copper addition decreased the intramuscular fat content by improving fatty acid uptake and fatty acid oxidation; (2) copper addition reduced the hepatic fat content by enhancing fatty acid oxidation; (3) copper addition inhibited lipid accumulation in adipocytes by promoting lipolysis and fatty acid oxidation; and (4) activated PPAR-α signaling in the liver, skeletal muscle, and adipose tissues and AMPK signaling in skeletal muscle tissue were involved in the reduction of intracellular fat content by copper.

### Copper promoted skeletal muscle growth and reduced excess fat accretion

In line with previous studies in ducks [17], laying hens [18], and broilers [34], copper treatment increased body weight gain and muscle tissue growth in rabbits. At the same time, copper treatment decreased liver and fat weight, suggesting an altered energy redistribution that favored lipolysis. Likewise, fat content analysis of liver, skeletal muscle, and adipose tissues suggested that copper treatment inhibited lipid accumulation in rabbits.

### Copper decreased the hepatic fat content by enhancing fatty acid oxidation

The liver plays a key role in lipid metabolism and is the hub of fatty acid synthesis and lipid circulation through lipoprotein synthesis in mammals. In line with previous studies in grass carp (22), copper treatment increased hepatic CPT gene expression [35]. The stimulation of CPT gene expression may reveal an increase in energy demand in the detoxification of reactive oxygen species produced by high copper level, as suggested by Stohs and Bagchi [36]. Thus, increased CPT1 and CPT2 mRNA levels indicated an increased lipolysis and a reduced lipid deposition in hepatocytes. However, in disagreement with previous studies in fish [24], copper treatment did not change the mRNA levels of lipogenic enzymes (ACC and FAS). The inconsistent results imply that copper regulation in hepatic lipogenesis differs in mammals and fish.

Hepatic lipid components that are synthesized in the smooth endoplasmic reticulum are glycosylated in the Golgi apparatus and released into the blood in the form of VLDL. The present study found that copper addition decreased the plasma VLDL concentration, which may be due to decreased substrate from the liver. Although copper treatment did not alter the hepatic anabolic pathway of fatty acids, it increased the catabolic pathway of fatty acids. The results suggest that copper decreased the production or output of triglycerides by the liver.

### Copper decreased the intramuscular fat content by enhancing fatty acid oxidation

Lipids are a major fuel source for oxidative metabolism, especially in cardiac and skeletal muscle [37]. However, the study of copper regulation of lipid metabolism in skeletal muscle is scarce. Several functional and structural steps are involved in lipid uptake, transport, and oxidation in muscle. FATP and FABP have been shown to facilitate long-chain fatty acid uptake and utilization in skeletal muscle [38]. In agreement with previous work in the intestine [16], the present study shows that the transcriptional levels of FATP and FABP increase following copper treatment (FATP, 1.5-fold; FABP, 1.35-fold), indicating that the capacity for fatty acid uptake by muscle cells is likely to be increased. The result is also related to the increased substrates (blood triglyceride concentration). In addition, copper improved fatty acid oxidation by upregulating CPT1 and CPT2 gene expression (CPT1, 3.6-fold; CPT2, 2.5-fold). Thus, skeletal muscle cells can obtain enough energy supply, improving skeletal muscle growth. Using Oil Red O staining, it was found that copper addition decreased the intramuscular fat content. The results implied that with the addition of copper, the speed of fatty acid transport and oxidation was greater than the uptake.

In addition, the partitioning of intracellular fatty acid from storage toward oxidation contributes to strengthening insulin sensitivity in skeletal muscle [38,39]. In the present study, copper supplement decreased plasma insulin levels, a finding that is consistent with the previous observation of copper deficiency–induced increases in pancreatic insulin [40]. The decreased insulin levels may be related to the increased sensitivity induced by copper through a decrease in the intramuscular triglycerlids content.

### Copper decreased the lipid content of adipose tissue by enhancing fatty acid oxidation and lipolysis

The effect of copper on lipid metabolism in adipose tissue remains unclear. We found that copper decreased adipose tissue weight, as well as the lipid content and size of adipocytes, which is associated with the enhanced process of fatty acid oxidation and lipolysis. LPL and HSL can hydrolyze extracellular and intracellular triglycerides in adipose tissue. In line with the work in the intestine [16], the present study showed that copper increased HSL gene expression in adipose tissue. This result suggests that copper can promote the hydrolysis of intracellular triglycerides [17,18]. In addition, copper improved fatty acid oxidation by upregulating CPT1 and CPT2 gene expression. Although the adipose tissue of young rodents can also synthesize lipid, copper treatment did not change the mRNA levels of lipogenic enzymes (ACC and FAS). Therefore, copper only promoted lipolysis but did not alter lipogenesis. The increased blood triglyceride concentration may result from lipolysis, and the increased circulating lipid ﬂux promotes fatty acid uptake and utilization by peripheral tissues such as muscles.

Leptin, an adipocytokine, may exert regulatory effects on the hypothalamus, liver, pancreatic islets, and skeletal muscle. Leptin functions as an afferent signal of a negative feedback loop to regulate body weight [41]. In the present study, copper treatment decreased plasma leptin levels with the decrease of adipose tissue. The latter signifies insufficient energy stores, promotes energy intake, reduces energy expenditure, and increases the partitioning of energy to fat, leading to a positive energy balance.

### The PPAR-α signaling pathway was involved in the regulation of lipid metabolism by copper

PPAR-α is known to regulate lipid metabolism through a ligand-dependent transcriptional activation of the expression of genes involved in the fatty acid oxidation pathway [42,43]. Hsu and Huang [42] reported that PPAR-α mRNA expression was positively correlated with mRNA expression of CPT1. The present study also indicated a similar tendency of PPAR-α expression and mRNA level of CPT1. Studies also suggest that PPAR-α stimulates through a peroxisome proliferator response element in the first and second intron of the human and rat CPT1 gene, respectively [44,45]. Thus, the present study indicated that copper addition influenced lipid metabolism in the liver, skeletal muscle, and adipose tissue, presumably through the PPAR-α signaling pathway.

PPAR-γ was initially characterized as the master regulator of the differentiation of fibroblast-like mesenchymal stem cells into adipocytes, a process known as adipogenesis [46]. The present study showed that copper did not alter the PPAR-γ mRNA level in adipose tissue, in agreement with a study of the liver [16]. Chen et al. [16] also found that PPAR-γ mRNA levels in adipose tissue only decreased with high doses and not with low doses of copper treatment, indicating that the effect of copper was related to dose. Zheng et al. [47] also indicated that PPAR-γ was positively related to FAS gene expression by zinc treatment. FAS gene expression was consistent with PPAR-γ in copper-treated rabbits. The present results also suggest that PPAR-γ is not a major target in the regulation of lipid metabolism by copper in adipose tissue.

### The AMPK signaling pathway was involved in the regulation of lipid metabolism by copper in skeletal muscle

AMPK, the metabolic sensor that monitors cellular AMP and ATP levels, is involved in the regulation of CPT1 [48] and has recently been described as an essential regulator of fatty acid oxidation in the skeletal muscle of mammals [49,50]. To account for the possibility that the changes in fatty acid oxidation were mediated by AMPK following copper treatment in rabbits, phosphorylation at Thr172 in the α-isoform of AMPK was measured because phosphorylation at this site by an upstream kinase is required for AMPK activity [51]. It was found that AMPK phosphorylation increased after copper treatment and that CPT gene expression increased in skeletal muscle. These results imply that the AMPK signaling pathway mediated the regulation of fatty acid oxidation by copper in skeletal muscle tissue but not in the liver or adipose tissue.

In conclusion, copper promoted skeletal muscle growth and reduced fat accretion. PPAR-α signaling in the liver, skeletal muscle, and adipose tissue and AMPK signaling in skeletal muscle tissue were involved in the regulation of lipid metabolism by copper.

## Supplementary Material

Supplemental_tables.zip
