# Supplementary material for: Effect of dietary copper addition on lipid metabolism in rabbits
Source: Food Nutr Res. 2017 Jul 6;61(1):1348866. doi: 10.1080/16546628.2017.1348866 (PMC5510220; doi:10.1080/16546628.2017.1348866)
Supplement: Supplemental_tables.zip [file zfnr_a_1348866_sm3353.zip › Supplemental tables/Supplemental Table S1.docx]

Supplemental Table S1 Composition and nutrient levels of basal diets (air-dry basis)

| Ingredients (%) | Content | Calculated chemical composition | Content |
| --- | --- | --- | --- |
| Corn | 7.00 | digestive energy (MJ/kg) | 10.28 |
| Wheat shorts | 16.00 | Crude protein % | 17.43 |
| Soybean meal | 8.00 | Crude fiber % | 19.81 |
| Germ | 15.00 | Crude fat % | 2.95 |
| Guinea grass | 20.00 | Ca % | 0.83 |
| Sunflower meal | 12.50 | P % | 0.35 |
| Husk powder | 13.00 |  |  |
| Expanded soybean | 4.00 |  |  |
| Bean oil | 0.75 |  |  |
| Phospholipid | 0.75 |  |  |
| Premix^*^ | 3.00 |  |  |
| Total | 100.00 |  |  |

^*^ The premix provided the following per kg of diets: VA 20000 IU, VD_3_ 2000 IU, VE 40 mg, VK_3_ 6 mg, VB_1_ 2 mg, VB_2_ 12 mg, VB_3_ 60 mg, VB_5_ 20 mg, VB_7_ 0.1 mg, VB_9_ 2 mg, VB_12_ 0.04 mg, choline chloride, 400 mg, Fe (as ferrous sulfate) 60 mg, Zn (as zinc sulfate) 60 mg, Mn (as manganese sulfate) 3 mg, I (as potassium iodide) 0.2 mg, Se (as sodium selenite) 0.2 mg, CaHPO_4_ 15 g, NaCl 5 g, Lys 1 g, Met 2 g, 10% bacitracin zinc 300 mg.
