# Supplementary material for: Effect of dietary copper addition on lipid metabolism in rabbits
Source: Food Nutr Res. 2017 Jul 6;61(1):1348866. doi: 10.1080/16546628.2017.1348866 (PMC5510220; doi:10.1080/16546628.2017.1348866)
Supplement: Supplemental_tables.zip [file zfnr_a_1348866_sm3353.zip › Supplemental tables/Supplemental Table S2.doc]

Supplemental Table S1 Gene-specific primers used for the analysis of rabbit genes expression

| Gene | Genebank Accession Number. | Primers sequences(5’→3’) | Product size(bp) |
| --- | --- | --- | --- |
| GAPDH | NM_001082253 | F: TGCCACCCACTCCTCTACCTTCG | 163 |
|  |  | R: CCGGTGGTTTGAGGGCTCTTACT |  |
| β-actin | NM_001101683.1 | F:CGCAGAAACGAGACGAGATT | 168 |
|  |  | R:GCAGAACTTTGGGGACTTTG |  |
| PPARα | XM_002723354 | F:AGGCCCTCTTCAGAACCTGT | 122 |
|  |  | R:GTGGCTTTCTGTTCCCAGAG |  |
| PPARγ | NM_001082148.1 | F:GGAGCAGAGCAAAGAAGTCG | 111 |
|  |  | R:CTCACAAAGCCAGGGATGTT |  |
| FATP | XM_002722970 | F:GGCCTACCTCTCTGGTGATG | 111 |
|  |  | R:TCAGTGGTGGACACGTTCTC |  |
| FABP | XM_002716060 | F:AGCTGGTGGACAGCAAGAAT | 129 |
|  |  | R:TCAGGGTGATGATGTCTCCA |  |
| CPT1 | XM_002724092.2 | F:ATTCTCACCGCTTTGGGAGG | 196 |
|  |  | R:ACGGGGTTTTCTAGGAGCAC |  |
| CPT2 | XM_008265231.1 | F:ATGACCGTTTCTGCCATCC | 101 |
|  |  | R: AAGGTGTTGGTGTCGCTTCT |  |
| FAS | KF201292.1 | F:ACCACGTCCAAGGAGAGCA | 112 |
|  |  | R:AGTTCTGCACCGAGTTGAGC |  |
| ACC | XM_002719077.2 | F:GTGGTCTTCGTGTGAACTGG | 122 |
|  |  | R:TTCTTCTGCTGCCTTTAGCC |  |
| HSL | XM_008249691.2 | F: CCAGGCTAAACTCGCATCCA | 119 |
|  |  | R: ATTTGGCTCTCTGGACTGGC |  |
| LPL | NM_001177330.1 | F: TTCAACCACAGCAGCAAGAC | 141 |
|  |  | R: TAACAGCCAGTCCACCACAA |  |

GAPDH, glyceraldehyde 3-phosphate dehydrogenase; PPAR, peroxisome proliferator-activated receptors; ACC, acetyl-CoA carboxylase; CPT, carnitine palmitoyltransferase 1; FAS, fatty acid synthase; HSL, hormone-sensitive lipase; LPL, lipoprotein lipase; FATP, fatty acid transport protein; FABP fatty acid transport protein.
